# Postural Tachycardia Syndrome and Vasovagal Syncope: A Hidden Case of Obstructive Cardiomyopathy without Severe Septal Hypertrophy

**DOI:** 10.1155/2018/8714819

**Published:** 2018-04-16

**Authors:** Kenneth A. Mayuga, Natalie Ho, Robert W. Shields, Paul Cremer, L. Leonardo Rodriguez

**Affiliations:** ^1^Department of Cardiovascular Medicine, Heart and Vascular Institute, Cleveland Clinic, Cleveland, OH, USA; ^2^Neurological Institute, Cleveland Clinic, Cleveland, OH, USA

## Abstract

A 36-year-old female with symptoms of orthostatic intolerance and syncope was diagnosed with vasovagal syncope on a tilt table test and with postural tachycardia syndrome (POTS) after a repeat tilt table test. However, an echocardiogram at our institution revealed obstructive cardiomyopathy without severe septal hypertrophy, with a striking increase in left ventricular outflow tract gradient from 7 mmHg at rest to 75 mmHg during Valsalva, with a septal thickness of only 1.3 cm. Cardiac MRI showed an apically displaced multiheaded posteromedial papillary muscle with suggestion of aberrant chordal attachments to the anterior mitral leaflet contributing to systolic anterior motion of the mitral valve. She underwent surgery with reorientation of the posterior medial papillary muscle head, resection of the tethering secondary chordae to the A1 segment of the mitral valve, chordal shortening and tacking of the chordae to the A1 and A2 segments of the mitral valve, and gentle septal myectomy. After surgery, she had significant improvement in her prior symptoms. To our knowledge, this is the first reported case of obstructive cardiomyopathy without severe septal hypertrophy with abnormalities in papillary muscle and chordal attachment, in a patient diagnosed with vasovagal syncope and POTS.

## 1. Introduction

Postural tachycardia syndrome (POTS) is a clinical syndrome characterized by frequent symptoms that occur with standing, an increase in heart rate of 30 bpm when moving from a recumbent to a standing position, and the absence of orthostatic hypotension [1]. Vasovagal syncope is defined as syncope that usually occurs with upright posture held for more than 30 seconds, features diaphoresis, warmth, nausea, and pallor, and is associated with hypotension and relative bradycardia [1]. Obstructive cardiomyopathy without severe septal hypertrophy (OCM), such as from papillary muscle abnormalities or abnormal chordal attachment, can lead to a decrease in cardiac output due to obstruction in the left ventricular outflow tract. To our knowledge, OCM from papillary muscle abnormalities and abnormal chordal attachment has not previously been reported in a patient diagnosed with POTS as well as vasovagal syncope.

## 2. Case Presentation

A 36-year-old female was referred to our institution for evaluation of symptoms of orthostatic intolerance. Consent was obtained from the patient to publish this as a case report, with documentation of verbal consent in the electronic medical record. She reported symptoms including dizziness, palpitations, nausea, and a warm sensation while upright that improved with assuming a recumbent posture. She also reported symptoms during exertion, documented as lightheadedness and feeling of her heart racing when physically active, as well as a decreased tolerance to walking. Her symptoms occurred a few times per week up to several times per day and were worse during the beginning of her menstrual cycle. Her symptoms significantly improved with wearing compression stockings. She also reported two episodes of loss of consciousness. With the first episode, she was lying on the couch then sat up and felt nauseated, and then she stood up and had an abrupt loss of consciousness. With the second episode, she was walking in the kitchen, had a brief prodrome of not feeling well, and then had an abrupt loss of consciousness. She had evaluations at an outside institution with a Holter monitor showing episodes of sinus tachycardia, an echocardiogram that was reportedly normal, and a tilt table test reporting a vasovagal reaction following the administration of nitroglycerin. She reported that her father had multiple episodes of syncope but never sought medical treatment; she did not report any family history of POTS.

She underwent further evaluations at our institution. A passive 70-degree head up tilt table test showed a postural increase in heart rate from 74 to 114 bpm at the end of the tilt, with some blood pressure oscillations but overall stable blood pressures (from 115/70 mmHg supine to 118/82 mmHg at the end of tilt) while eliciting her typical symptoms of nausea, warmth, a spinning sensation, and feeling like she would lose consciousness. She requested the test to be stopped early at 36 minutes out of a 45-minute protocol due to her symptoms. Of note, she was taking fludrocortisone 0.1 mg daily at the time of the head up tilt table test. She was diagnosed initially with POTS due to her clinical features and tilt table test results. She underwent blood volume and hemodynamic testing. Blood volume measured using a I-131-HSA-tagged human serum albumin technique with a Daxor BVA-100 system (Daxor Corporation, NY, USA) showed normal plasma volume at −2.8% from ideal (gender matched, weight, and height). Hemodynamic testing to assess circulatory kinetics was done using 99 m Technetium-RBC radionuclide imaging [2]. At 45-degree head up position, the heart rate increased by 18 bpm, cardiopulmonary volume fraction decreased by 34% (a marked decrease), cardiac index decreased from 2.6 L/min/m^2^ to 1.777 L/min/m^2^, stroke volume decreased from 79 ml to 50 ml, total peripheral resistance increased from 40 to 61 *μ*m^2^, and mean pulmonary transit time was rapid at 6.8 seconds. Plasma metanephrine and normetanephrine levels were checked and were within normal limits. An evaluation of autonomic function was done. Quantitative sudomotor axon reflex testing showed normal values at the right forearm, proximal leg, distal leg, and foot. The heart rate and blood pressure responses to Valsalva showed a normal Valsalva ratio of 1.47 and normal blood pressure responses in phase II and phase IV. An exercise treadmill test done as part of the evaluation for cardiac rehabilitation showed fair functional capacity at 8.6 METS, an increase in heart rate to 99% of maximal predicted heart rate, stable blood pressures, and no arrhythmias.

**Figure 1:**
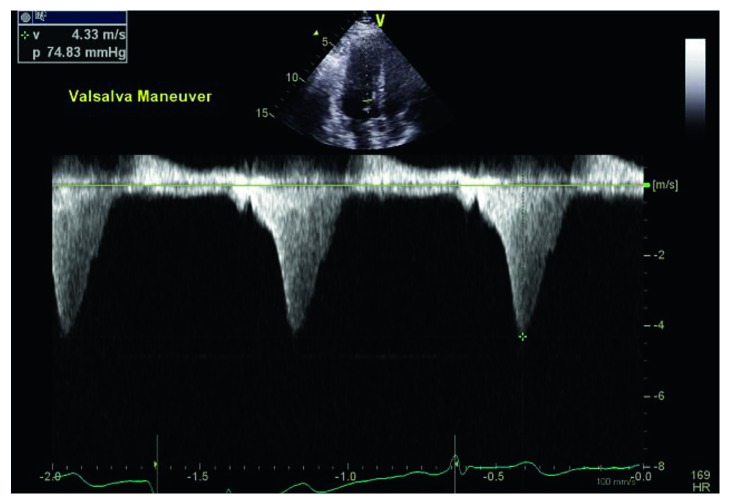
Echocardiogram showing 75 mmHg gradient in the left ventricular outflow tract with Valsalva.

**Figure 2:**
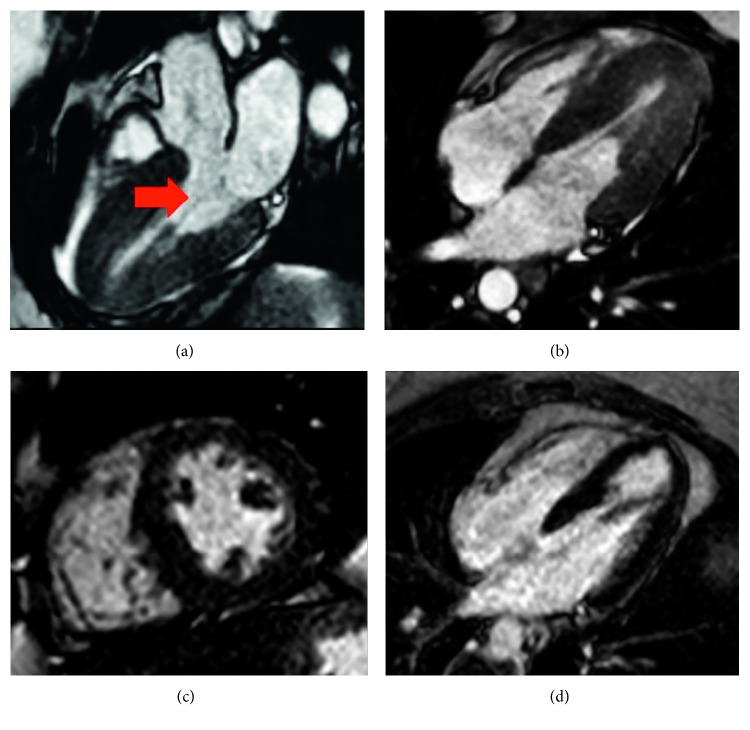
Cardiac MRI showing multiple heads of the posteromedial papillary muscle in the three chamber view (a), four chamber view (b), and short axis view (c), with a suggestion of an abnormal chordal attachment to the anterior mitral valve leaflet (red arrow). Delayed gadolinium imaging (d) showing no increased signal.

**Figure 3:**
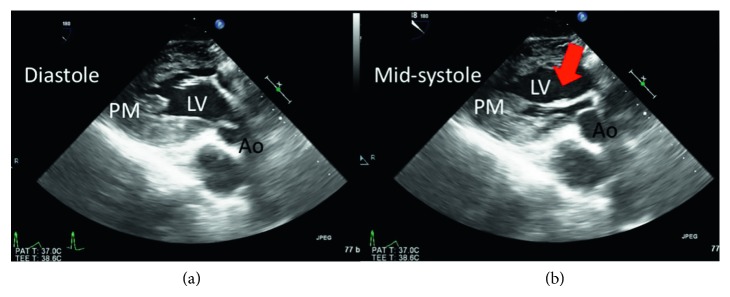
Long axis of the LV from a deep transgastric view using transesophageal echocardiography. LV = left ventricle, PM = posterior papillary muscle, and Ao = aorta. During midsystole, there is anterior motion of the apically displaced posterior papillary muscle (red arrow).

A resting echocardiogram repeated at our institution showed a normal EF of 71%, mild septal left ventricular hypertrophy measuring 1.3 cm, with a prominent posterior papillary muscle. At rest, there was chordal systolic anterior motion (SAM) of the mitral valve with trivial mitral regurgitation, and a left ventricular outflow tract (LVOT) gradient of only 7 mmHg. After Valsalva maneuver, the LVOT gradient increased strikingly to 75 mmHg (Figure 1) with a further increase to 81 mmHg after administration of amyl nitrite. A subsequent cardiac MRI demonstrated an apically displaced, multiheaded posteromedial papillary muscle. There was a suggestion that the more anteriorly displaced head of the posteromedial papillary muscle had aberrant chordal attachments to the anterior mitral valve leaflet that contributed to the SAM (Figure 2). There was mild hypertrophy of the anteroseptum (measuring 1.3 cm) with a normal left ventricular mass index of 57 gm/m^2^ (normal 48–77 gm/m^2^) and no delayed enhancement to suggest interstitial fibrosis. A subsequent transesophageal echocardiogram (TEE) demonstrated the mobile anteriorly displaced posterior papillary muscle with SAM (Figure 3).

After consultations with specialists in structural heart disease and cardiothoracic surgery, the patient ultimately underwent cardiac surgery consisting of very gentle septal myectomy focusing more midventricular at the basilar septum, reorientation of the posterior medial papillary muscle head, resection of the tethering secondary chordae to the A1 segment of the mitral valve, and chordal shortening and tacking of the chordae to the A1 and A2 segments of the mitral valve. During her several post-op visits with Cardiology and Cardiothoracic Surgery, she reported a significant improvement in her prior orthostatic symptoms and no longer reported syncope.

## 3. Discussion

Obstructive cardiomyopathy without severe septal hypertrophy, for example, from papillary muscle abnormalities or abnormal chordal attachment, is not as recognized as the more widely known hypertrophic obstructive cardiomyopathy with severe septal hypertrophy (HOCM) but can also lead to symptoms due to left ventricular outflow obstruction and decreased cardiac output. In the case presented, the patient underwent typical evaluations for a patient with orthostatic symptoms, syncope, and POTS, with expected findings on the initial evaluations, including a tilt table test. Typically, echocardiograms are done while the patient is at rest and without provocative maneuvers such as Valsalva or amyl nitrite unless a resting LVOT gradient is seen. Therefore, entities such as OCM can be readily underdiagnosed. The repeat echocardiogram at our institution disclosed no significant LVOT gradient at rest, but it did reveal a prominent posterior papillary muscle and mild septal hypertrophy. Valsalva maneuver demonstrated significant obstruction across the LVOT, with similar findings after administration of amyl nitrite, a vasodilator agent. The patient also underwent additional cardiac imaging with TEE and MRI that demonstrated the apically displaced, multiheaded posteromedial papillary muscle with SAM. Initial treatment with a beta-blocker can be considered to slow the heart rate and enhance left ventricular filling, but in some cases surgical correction is considered, as in this case. The diagnosis and treatment of obstructive cardiomyopathy without severe hypertrophy (≤1.8 cm) has recently been reported from our institution [3]. On multivariate analysis, the predictors of maximal LVOT gradient included bifid papillary muscle mobility and abnormal chordal attachment to the base of the anterior mitral leaflet. In terms of management, it is important to differentiate patients with OCM as opposed to HOCM. As opposed to the surgical treatment of HOCM, which involves myectomy to relieve obstruction, performing myectomy in patients with OCM may not effectively relieve the obstruction. Patel et al. [3] showed that >50% of the patients with OCM required a non-myectomy approach to optimally relieve LV outflow tract obstruction, such as with papillary muscle reorientation. Newer techniques such as this have an added benefit of decreasing the need for mitral valve replacement for these patients.

In this case, given the venous pooling found on hemodynamic testing, a decrease in venous return to the heart with upright posture can provoke or worsen an LVOT gradient, resulting in reduced cardiac output, and subsequently causing symptoms. The hemodynamic testing indeed showed a decrease in the cardiac index when the patient was tilted to 45 degrees, even with an increase in the heart rate, due to a decrease in stroke volume. This may help explain the significant benefit that this patient derived from wearing lower extremity compression stockings with the limiting of venous pooling. Overall, this case illustrates the importance of maintaining a high degree of suspicion when assessing for cardiac etiologies, including considering the use of provocative maneuvers or multimodality cardiac imaging, in patients with otherwise presumed typical POTS and vasovagal syncope. To our knowledge, this is the first reported case of obstructive cardiomyopathy without severe septal hypertrophy with abnormalities in papillary muscle and chordal attachment, in a patient diagnosed with POTS and vasovagal syncope.

## Abbreviations

POTS: Postural tachycardia syndrome
OCM: Obstructive cardiomyopathy without severe septal hypertrophy
HOCM: Hypertrophic obstructive cardiomyopathy
SAM: Systolic anterior motion
LVOT: Left ventricular outflow tract.
